# Efficacy and safety of intravenous acetaminophen (2 g/day) for reducing opioid consumption in Chinese adults after elective orthopedic surgery: A multicenter randomized controlled trial

**DOI:** 10.3389/fphar.2022.909572

**Published:** 2022-07-22

**Authors:** Feng Yin, Wei Ma, Qiao Liu, Liu-Lin Xiong, Ting-Hua Wang, Qian Li, Fei Liu

**Affiliations:** ^1^ Department of Anesthesiology, West China Hospital, Sichuan University, Chengdu, Sichuan, China; ^2^ Department of Anesthesiology, Chenzhou No. 1 People’s Hospital, Chenzhou, Hunan, China; ^3^ Institute of Neurological Disease, Translational Neuroscience Centre, West China Hospital, Sichuan University, Chengdu, Sichuan, China; ^4^ Institute of Neuroscience, Kunming Medical University, Kunming, Yunnan, China

**Keywords:** intravenous acetaminophen, orthopedic surgery, morphine consumption, opioid-sparing, multimodal analgesia

## Abstract

**Background:** Acetaminophen is an important component of a multimodal analgesia strategy to reduce opioid consumption and pain intensity after an orthopedic surgery. The opioid-sparing efficacy of intravenous acetaminophen has been established at a daily dose of 4 g. However, it is still unclear for the daily dose of 2 g of acetaminophen, which is recommended by the China Food and Drug Administration Center for Drug Evaluation, in terms of its efficacy and safety.

**Objectives:** This study aimed to evaluate the efficacy and safety of intravenous acetaminophen at a daily dose of 2 g for reducing opioid consumption and pain intensity after orthopedic surgery.

**Methods:** In this multicenter, randomized, double-blind, placebo-controlled phase III trial, 235 patients who underwent orthopedic surgery were randomly assigned to receive intravenous acetaminophen 500 mg every 6 h or placebo. Postoperative morphine consumption, pain intensity at rest and during movement, and adverse events were analysed.

**Results:** For the mean (standard deviation) morphine consumption within 24 h after surgery, intravenous acetaminophen was superior to placebo both in the modified intention-to-treat analysis [8.7 (7.7) mg vs. 11.2 (9.2) mg] in the acetaminophen group and the placebo group, respectively. Difference in means: 2.5 mg; 95% confidence interval, 0.25 to 4.61; *p* = 0.030), and in the per-protocol analysis (8.3 (7.0) mg and 11.7 (9.9) mg in the acetaminophen group and the placebo group, respectively. Difference in means: 3.4 mg; 95% confidence interval: 1.05 to 5.77; *p* = 0.005). The two groups did not differ significantly in terms of pain intensity and adverse events.

**Conclusion:** Our results suggest that intravenous acetaminophen at a daily dose of 2 g can reduce morphine consumption by Chinese adults within the first 24 h after orthopedic surgery, but the extent of reduction is not clinically relevant.

**Clinical Trial Registration**: [ClinicalTrials.gov], identifier [NCT02811991].

## 1 Introduction

Opioids are the cornerstone treatments against postoperative pain. However, it also associates with adverse effects such as nausea, vomiting, respiratory depression and risk of addiction (Wheeler et al., 2002; Rian et al., 2021). Multimodal analgesia has emerged as an approach capable of alleviating postoperative pain, shortening hospitalization, enhancing recovery, and increasing the satisfaction extent of patients while reducing the opioid use and therefore opioid-related adverse events (Artime et al., 2018; Olbrecht et al., 2018; Tong et al., 2018; Horsley et al., 2019; Marcotte et al., 2020; Mujukian et al., 2020). One of the oldest, most frequently used non-opioid analgesics is acetaminophen, which has few contraindications and shows relatively little side-effects when being used clinically (Lee et al., 2019). Administering acetaminophen intravenously may be better than delivering it orally or rectally because it bypasses the gastrointestinal tract and therefore can more quickly achieve maximal concentration in the central nervous system (Singla et al., 2012). Intravenous administration of acetaminophen was approved by the US Food and Drug Administration in 2010 (Lachiewicz, 2013).

Given the hepatotoxicity of acetaminophen, the US Food and Drug Administration recommends a maximum daily dose of 4 g acetaminophen for adults and adolescents weighing at least 50 kg (Richette et al., 2015). In European Union, a maximum dose of 3 g daily is recommended for older individuals whose body weight are bigger than 50 kg and are under certain risk for hepatotoxicity (Mian et al., 2018). In contrast, the Chinese Center for Drug Evaluation recommends a maximum daily dose of 2 g for all adults (Evaluation and Considerations regarding the dosage of acetaminophen, 2012), likely because of the adverse events linked to acetaminophen dosage such as hepatotoxicity (Lee et al., 2015; Seifert et al., 2016), as well as the lower body mass index of Chinese population than other ethnic groups (NCD-RisCNRFC, 2017).

The opioid-sparing efficacy of intravenous acetaminophen has been well established at a daily dose of 4 g in patients undergoing total hip arthroplasty, knee arthroplasty or other orthopedic surgeries (Peduto et al., 1998; Hernández-Palazón et al., 2001; Sinatra et al., 2005; Gupta et al., 2016; Camu et al., 2017; Thybo et al., 2019). However, the efficacy and safety of intravenous acetaminophen at a daily dose of 2 g remains unclear despite that it is recommended by the China Food and Drug Administration Center for Drug Evaluation. Thus, we conducted a multicenter, randomized, placebo controlled, phase III trial to investigate the efficacy and safety of intravenous acetaminophen at a daily dose of 2 g in patients who underwent orthopedic surgery.

## 2 Methods

### 2.1 Study design

This multicenter, randomized, double-blinded, placebo-controlled phase III trial was conducted between 14 August 2017 and 4 September 2018 at 17 centers across China (Supplementary Appendix I), and the trial management at each center is described in Supplementary Appendix II. This trial was funded by Jiangsu Heng Rui Medicine Co., Ltd. (Jiangsu, China), and was approved by the State Food and Drug Administration of China (permit 2015L04750) as well as the Ethics Committees of all participating institutions. The trial protocol followed the “Standard Protocol Items: Recommendations for Interventional Trials” (SPIRIT) guidelines (Chan et al., 2013) and was conducted in accordance with the International Conference on Harmonization Guidelines for Good Clinical Practice (ICH-GCP) and the Declaration of Helsinki (Baber, 1994). All study subjects provided written informed consents prior to enrollment. The trial was registered with ClinicalTrials.gov (NCT02811991) in June 2016, enrollment began in August 2017, and results here are reported according to the “Consolidated Standards of Reporting Trials” (CONSORT) guidelines (Schulz et al., 2010).

### 2.2 Inclusion and exclusion criteria

Patients were potentially eligible for the trial if they 1) were between 18 and 70 years old; 2) were scheduled for major orthopedic surgery under general anesthesia during the enrollment period; 3) had an American Society of Anesthesiologists physical status of I or II; 4) had a body mass index of 18–30 kg/m^2^; and 5) were able to understand the pain scales and operate an intravenous patient-controlled analgesia (PCA) device.

Patients were excluded if they had 1) contraindications to the study medications; 2) uncontrolled hypertension or diabetes; 3) having known history of pulmonary or heart disease; 4) impaired liver function, defined as alanine aminotransferase and/or aspartate aminotransferase levels are more than 2 folds higher than the upper limit of the normal range, or the total bilirubin is at least 1.5 times as the upper limit of the normal range; 5) renal dysfunction, defined as the creatinine level >176 μmol/L. Patients were also excluded from the trial if they were 6) pregnant or 7) at high risk of bleeding.

### 2.3 Randomization and blinding

After screening, patients who were eligible to the trial were randomly assigned to one of the two groups at 1:1 ratio: the acetaminophen group and the placebo group. The “acetaminophen group” received 500 mg intravenous acetaminophen every 6 h for 24 h, while the “placebo group” received the same volume of saline every 6 h for 24 h. Randomization was performed by statisticians using an interactive, web-based randomization system (College of Public Health, Nanjing Medical University, Nanjing, China). The randomization sequence was created in SAS (version 9.4, SAS Institute, Cary, NC, United States) with 1:1 allocation and a fixed block size of 4. Each patient received a unique code of randomization and a medication bottle. The statisticians sent group allocation information directly to the central pharmacy (Jiangsu Heng Rui Medicine Co., Ltd.), which prepared infusion bottles containing 100 ml of acetaminophen (1,000 mg) or the same volume of saline as placebo labelled with a unique code. A single infusion dose of 50 ml was also labelled on the infusion bottles. The appearance and packaging of the bottles for two groups were identical. Bottles were shipped to each study site, then distributed to independent investigators. Thus, investigators, patients, surgeons and clinical personnel were blinded to group allocation.

### 2.4 General anesthesia

Surgery was performed under general anesthesia, which was induced with propofol (2–4 mg/kg), midazolam (2 mg), sufentanil (0.3–0.5 ug/kg) and cisatracurium (0.2 mg/kg) after pure oxygen inhalation. Anesthesia was maintained continuous by infusion of sevoflurane at a minimal alveolar concentration of 1–1.5 and remifentanil at 0.1–0.3 µg/kg/min. Sufentanil was allowed to be added if required. All medications for intraoperative analgesia were discontinued before the first dose of the study drug was given. In addition, sufentanil and other long-acting analgesics were discontinued at least 15 min prior to the start of the first dose of the study drug. Tropistron was used at 30 min before the end of surgery in order to prevent postoperative nausea and vomiting. Patients received either 50 ml acetaminophen (500 mg) or 50 ml placebo through 15–30 min intravenous infusion. The infusion was repeated every 6 h for a total of four times.

### 2.5 Postoperative care

During the first 24 h after surgery, patients received an intravenous PCA device to self-administer morphine; the drug concentration was 0.2 mg/ml; the bolus volume was 1 mg; the lock-out time was 5 min; and the background infusion rate was 0.5 ml/h. If pain was not relieved using this device, a 2-mg bolus of intravenous morphine was allowed for rescue analgesia. Such rescue analgesia was included in the 24-h morphine consumption (see next section).

### 2.6 Assessments and outcomes

On the day before surgery, patients were instructed for how to use the PCA pump and numeric rating scale (NRS), which ranged from 0 (no pain) to 10 (worst possible pain), by one of the investigators. Patients were also be educated to press the button of PCA if they felt pain.

All postoperative assessments were performed by a trained doctor. Cumulative PCA intravenous morphine consumption was recorded during the first 24 h after surgery. Pain was evaluated with the NRS both at rest and during movement (stretching the corresponding joint) at 1, 3, 6, 12, and 24 h postoperatively. Adverse events were evaluated during the first 72 h after surgery. An adverse event was defined as any untoward medical occurrence in the patient, regardless of whether it was likely caused by the study medication. Serious adverse events were defined as adverse events that posed substantial risk of death, prolongation of hospitalization or rehospitalization, or that caused substantial or persistent disability or incapacity according to ICH-GCP guidelines (Baber, 1994). Adverse events were detected based on clinical observations, vital sign recording, 12-lead electrocardiography and laboratory examinations. Routine laboratory examinations of blood, urine, as well as liver and kidney function were performed at enrollment and 24–72 h postoperatively. Adverse events were attributed to the acetaminophen based on the criteria in Supplementary Appendix III.

The primary outcome was morphine consumption within 24 h after surgery. Secondary outcomes included pain intensity at rest and during movement at 1, 3, 6, 12, and 24 h postoperatively, the area under the pain intensity-time curve (AUC_1–24h_, AUC_6–24h_, and AUC_12–24h_) for pain at rest and during movement, as well as the number of total and effective PCA button press during the first 24 h postoperatively. The safety outcome was evaluated by the incidence of adverse events.

### 2.7 Sample size calculation and statistical analysis

A pilot study, detailed in Supplementary Appendix IV, indicated that the mean (standard deviation [SD]) difference between the acetaminophen and placebo groups in terms of the morphine consumption amount during the first 24 h postoperatively was 5.0 (10.0) mg. In order to detect such difference, the minimal number of patients in each group predicted by the PASS software (version 15; NCSS, Kaysville, UT, United States) was 80, assuming a type 1 error of 0.05 and the power of 80%. Given the expected dropout rate of 15%, the number of required patients enrolled in each group was 120.

Statistical analysis was conducted following the modified intention-to-treat principle (Le Henanff et al., 2006), meaning that all randomized patients who received at least one administration of the study medication and completed at least one NRS measurement were included. The per-protocol population included patients who met the eligibility criteria, completed the treatment without any major violation of the study protocol, and for whom primary and secondary outcomes could be analyzed.

Continuous data were presented as mean (SD) or median (interquartile range, IQR) as appropriate. Data normality was tested using the Shapiro-Wilk test. Differences were assessed for significance using Student’s t test or Mann-Whitney U test. Categorical data were presented as number (percentage), and differences were assessed for significance using Pearson’s χ^2^ test or Fisher’s exact test, as appropriate. In some cases, results were also presented using 95% confidence intervals (CIs). Generalized estimating equations with robust standard error estimates were used to correct repeated measurement of pain scores.

All analyses were performed using SAS 9.4 (The SAS Institute, Cary, NC, United States). Differences associated with two-sided *p* < 0.05 were considered statistically significant.

## 3 Results

### 3.1 Patient characteristics

**FIGURE 1:**
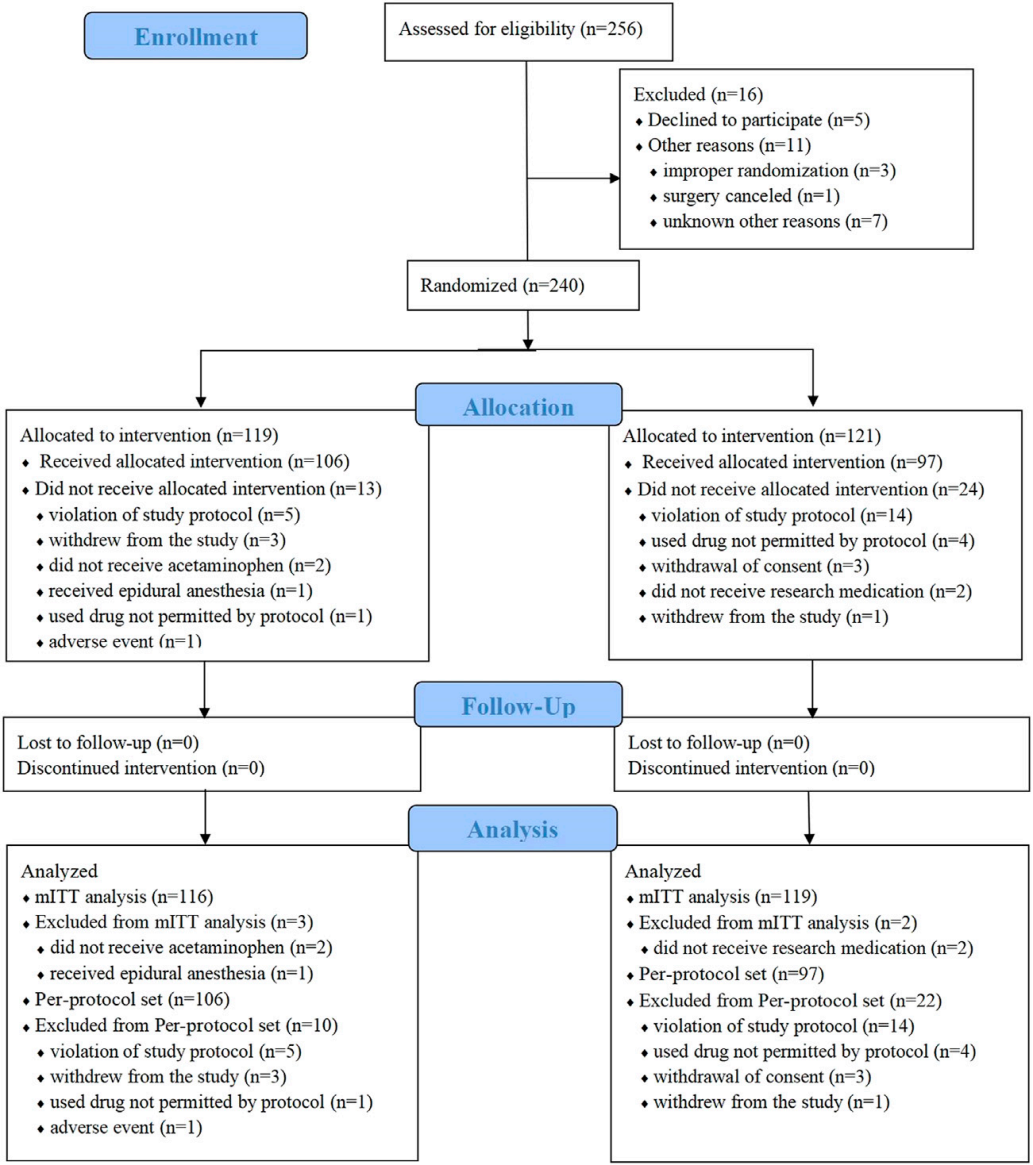
Flow CONSORT diagram of study recruitment. mITT, modified intention-to-treat.

A total of 272 patients were screened for eligibility between 14 August 2017 and 4 September 2018 (Figure 1). Among them, 32 patients were excluded including 16 patients due to their failure to meet the eligibility criteria, 5 patients due to their denying to participate, 3 patients due to the failure of randomization, and 1 patient due to the cancelled surgery, and 7 patients for other reasons. In the end, 240 patients were enrolled, of whom 119 were randomized into the acetaminophen group and 121 into the placebo group.

In the acetaminophen group, 2 patients did not receive the study medication, and 1 patient received epidural anesthesia instead of general anesthesia. As a result, the modified intention-to-treat population in this group was 116 patients. In the placebo group, 2 patients withdrew from the study, so the modified intention-to-treat population was 119 patients.

The two groups were similar in terms of sex distribution, age, weight, height, body mass index, and American Society of Anesthesiologists physical status and surgical information (Table 1).

**TABLE 1:** Baseline patient characteristics and perioperative data^a^.

Characteristic	Acetaminophen (*n* = 116)	Placebo (*n* = 119)
Patient characteristics		
Female, n/total N (%)	49/116 (42%)	53/119 (45%)
Age (yr), mean (SD)	49.6 (12.6)	50.3 (12.8)
Weight (kg), mean (SD)	63.7 (11.7)	64.8 (9.4)
Height (m), mean (SD)	1.6 (0.1)	1.6 (0.1)
Body mass index (kg/m^2^), mean (SD)	23.7 (3.1)	24.0 (2.7)
Perioperative data, mean (SD)		
Duration of surgery (min)	136.0 (65.2)	126.1 (59.2)
Mean heart rate (per min)	76.3 (11.1)	77.3 (10.1)
Mean systolic blood pressure (mmHg)	126.1 (12.9)	126.7 (12.6)
Mean diastolic blood pressure (mmHg)	78.4 (8.8)	79.8 (9.1)
ASA status, n/total N (%)		
I	25/116 (22%)	40/119 (34%)
II	91/116 (78%)	79/119 (66%)
Type of surgery, n/total N (%)		
Total knee or hip arthroplasty	25/116 (22%)	29/119 (24%)
Posterior internal fixation cervical/thoracic/lumbar vertebra	23/116 (20%)	18/119 (15%)
Long bone fracture	18/116 (16%)	20/119 (17%)
Others	50/116 (43%)	52/119 (44%)

SD, standard deviation; ASA, american society of anaesthesiologists.

a The two groups did not differ significantly on any of the characteristics shown.

### 3.2 Primary outcome

The patients in acetaminophen group consumed significantly less morphine than the placebo group during the first 24 h after surgery (Table 2). This result was obtained both in the modified intention-to-treat analysis and per-protocol analysis. The results from the modified intention-to-treat analysis were as the following: the mean amount of consumed morphine was 8.7 (7.7) mg in the acetaminophen group compared to 11.2 (9.2) mg in the placebo group. The difference in mean values was 2.5 mg, and the 95% confidence interval was 0.25–4.61, with a *p* value of 0.03. The results from the per-protocol analysis demonstrated that the consumed morphine was 8.3 (7.0) mg and 11.7 (9.9) mg in the acetaminophen group and the placebo group, respectively. The difference in mean values was 3.4 mg, the 95% confidence interval was 1.05–5.77, with a *p* value of 0.005.

**TABLE 2:** Cumulative morphine consumption (mg) within the first 24 h after surgery.

Analysis mehtod	Acetaminophen (*n* = 116)	Placebo (*n* = 119)	*p*
Modified intention-to-treat, mean (SD)	8.7 (7.7)	11.2 (9.2)	0.03
Per-protocol, mean (SD)	8.3 (7.0)	11.7 (9.9)	0.005

SD, standard deviation.

### 3.3 Secondary outcomes

**FIGURE 2:**
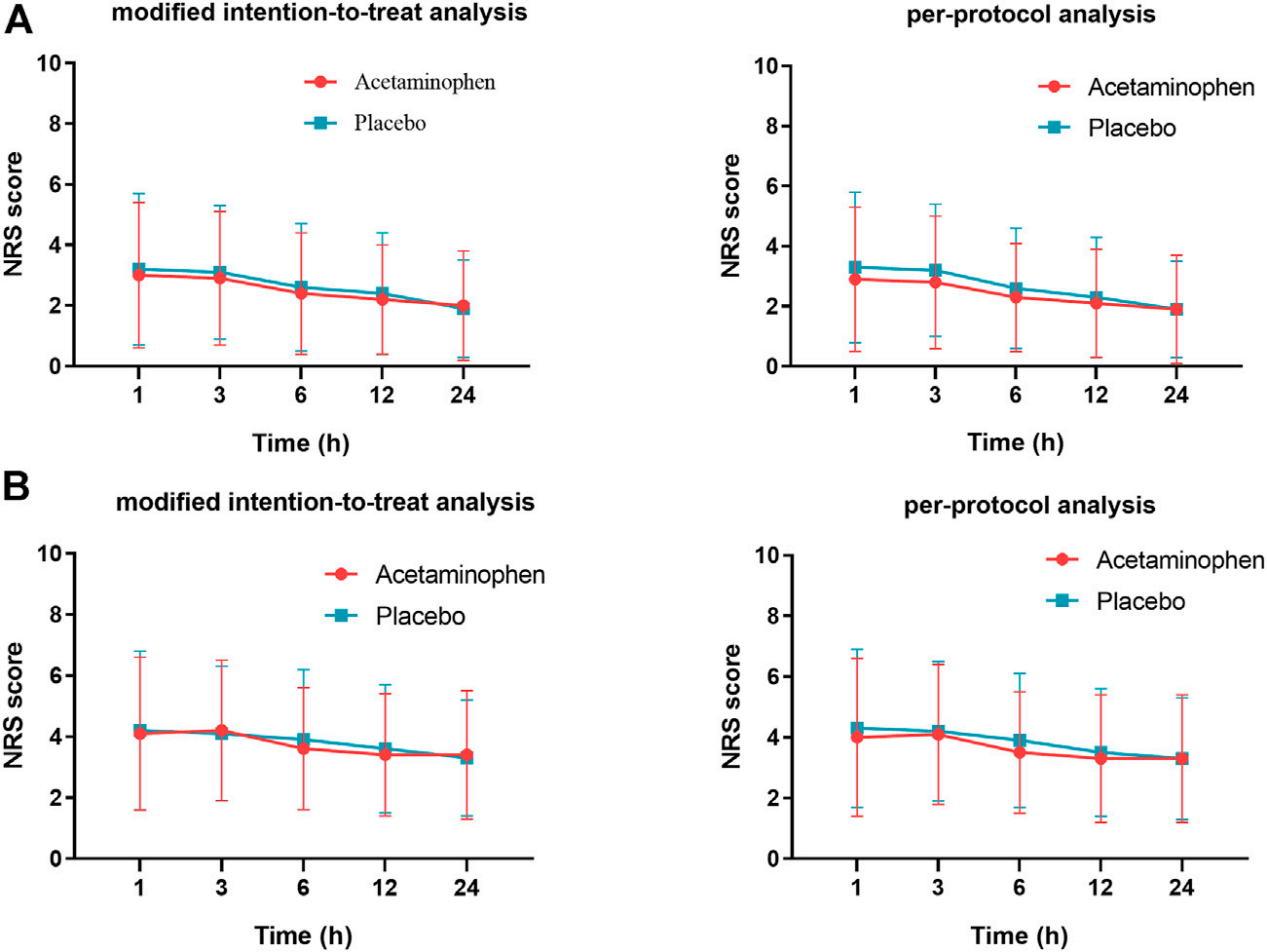
Intensity of pain at rest **(A)** and during movement **(B)** during the first 24 h after surgery. There is no significant difference between the acetaminophen group and placebo group (*p* > 0.05). NRS, numerical rating scale.

**FIGURE 3:**
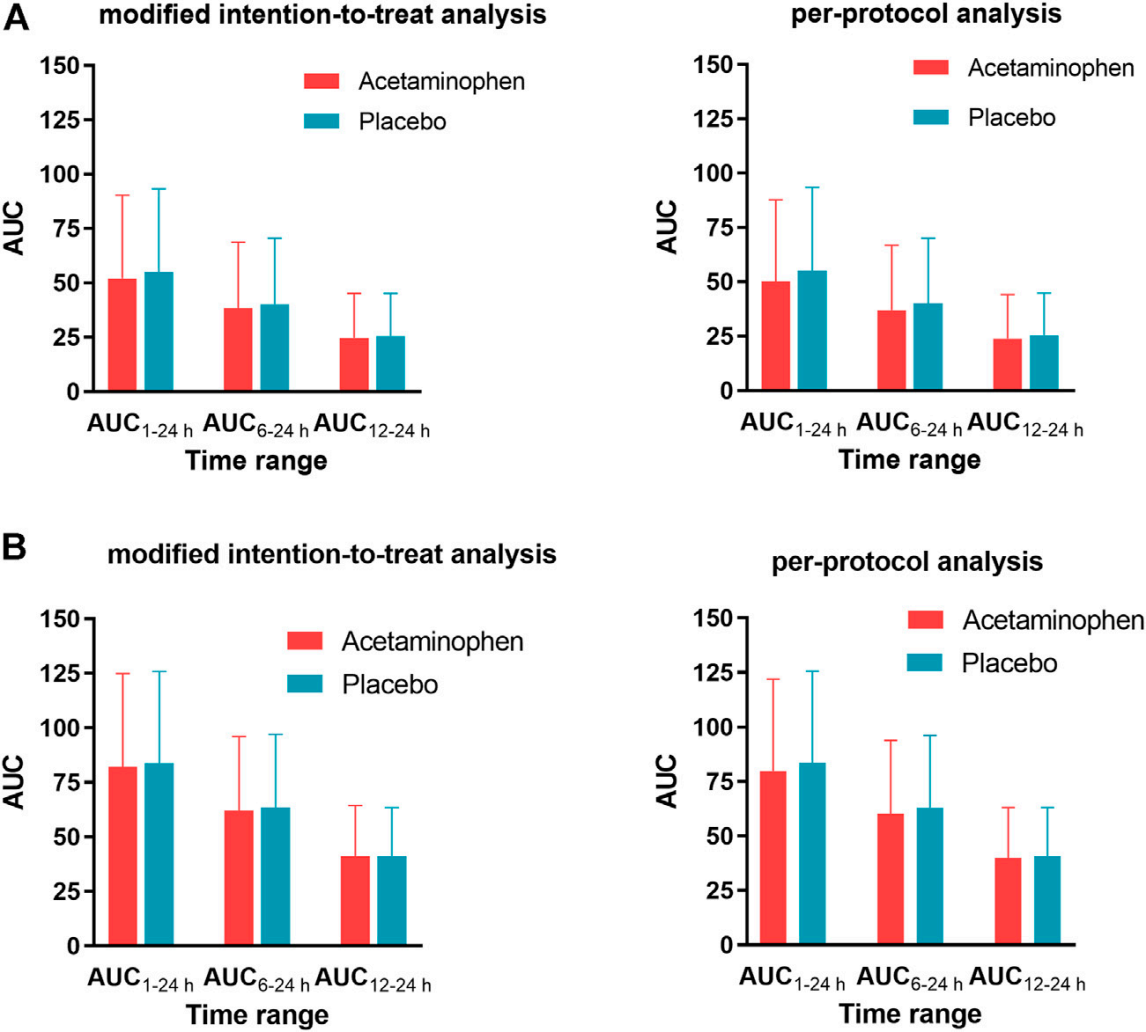
Area under the curve (AUC) of pain intensity-time at rest **(A)** or during movement **(B)**. The two groups did not differ significantly for any AUC (*p* > 0.05).

The pain intensity in the two groups did not differ significantly (Figure 2), nor did the AUC_1–24h_, AUC_6–24h_, or AUC_12–24h_ of pain (Figure 3) at rest or during movement. This result was consistent in both the modified intention-to-treat analysis and the per-protocol analyses.

In terms of the times of button-press on PCA device, the results from the per-protocol analysis showed that patients in the acetaminophen group pressed the button less than those in the placebo group during the first 24 h (4 (interquartile range 1–10) and 6 (interquartile range 2–13) in acetaminophen group and placebo group, respectively, *p* = 0.02). So did the times of button-press that resulted in effective analgesia (4 (interquartile range 1–8) in the acetaminophen group; 6 (interquartile range, 2–13) in the placebo group, *p* = 0.01) (Table 3). But there was no significant difference observed when the data was analysed under the intention-to-treat analysis regarding the total button-press times and the button-press times leading to effective analgesia.

**TABLE 3:** Patient use of controlled analgesia pump during the first 24 h after surgery.

Variables	Acetaminophen (*n* = 116)	Placebo (*n* = 119)	*p* value
Times of button-pressing			
Modified intention-to-treat analysis, median [IQR]	4.0 (1.0–11.0)	6.0 (2.0–13.0)	0.10
Per-protocol analysis, median [IQR]	4.0 (1.0–10.0)	6.0 (2.0–13.0)	0.02
Times of effective button-pressing			
Modified intention-to-treat analysis, median [IQR]	4.0 (1.0–9.0)	6.0 (2.0–13.0)	0.07
Per-protocol analysis, median [IQR]	4.0 (1.0–8.0)	6.0 (2.0–13.0)	0.01

IQR, interquartile range.

### 3.4 Safety outcomes

Rates of all adverse events or serious adverse events did not differ significantly between the two groups (Table 4). The incidence of adverse events was 83/116 (72%) in the acetaminophen group and 87/119 (73%) in the placebo group (*p* = 0.77). The incidence of severe adverse events was 3/116 (3%) in the acetaminophen group and 1/119 (1%) in the placebo group (*p* = 0.37). Only one patient in the acetaminophen group demonstrated a moderate increase of alanine aminotransferase and aspartate aminotransferase activities on the fourth day after surgery, and the activities of these enzymes gradually returned to normal levels later on.

**TABLE 4:** Adverse events in the study^a^.

Event	Acetaminophen (*n* = 116)	Placebo (*n* = 119)	*p* value
Severe adverse events, n/total N (%)	3/116 (3%)	1/119 (0.8%)	0.37
Postoperative infection	1/116 (0.9%)	0/119 (0%)
Scrotal hematoma	1/116 (0.9%)	0/119 (0%)	
Reperfusion-induced injury	1/116 (0.9%)	0/119 (0%)
Intraoperative hypotension	0/116 (0%)	1/119 (0.8%)	
Adverse events, n/total N (%)			
Fever	31/116 (27%)	39/119 (33%)	0.32
Postoperative nausea and vomiting	25/116 (22%)	28/119 (24%)	0.70
Anemia	17/116 (15%)	20/119 (17%)	0.72
Dizziness	8/116 (7%)	10/119 (8%)	0.81
Hypoproteinemia	6/116 (5%)	9/119 (8%)	0.60
Decreased appetite	6/116 (5%)	9/119 (8%)	0.60

a Only adverse events occurring in at least 5% of patients in either group are shown.

## 4 Discussion

We have demonstrated that intravenous administration of 500 mg acetaminophen every 6 h in 24 h postoperatively reduced the supplemental intravenous morphine consumption in Chinese adults who underwent an elective orthopedic surgery, and the reduction was statistically significant. This is the first study to assess the efficacy and safety of intravenous acetaminophen at a daily dose of 2 g for reducing postoperative pain and opioid consumption. Reducing opioid consumption and related complications has become particularly important (Gabriel et al., 2019) given the disturbing increases of opioid-use disorders and opioid-related mortality that result from an explosion of the opioids prescription (Chou et al., 2015; Volkow and McLellan, 2016).

Although acetaminophen, as one of the most commonly used medication for postoperative pain relief, the analgesic mechanism of the medication is not definitively known. Some study results indicate that acetaminophen functions as an analgesic agent via inhibiting cyclooxygenase pathway and reducing the production of prostaglandins (Graham and Scott, 2005; Smith, 2009; Candido et al., 2017). Others reported that acetaminophen could reinforce the descending inhibitory pain pathways mediated by 5-hydroxytryptamine (Pickering et al., 2008).

Data from numerous studies have established the efficacy of intravenous acetaminophen at dose level of 4 g daily to reduce the 24-h opioid consumption after total hip arthroplasty, knee arthroplasty and other types of orthopedic surgery (Delbos and Boccard, 1995; Sinatra et al., 2005; Camu et al., 2017; Huang et al., 2018; Takeda et al., 2019). The decreased morphine consumption could minimize the adverse effects of opioids, leading to an earlier postoperative ambulation, returning of normal bowel function, and earlier postoperative discharge (Marcotte et al., 2020). It is unclear whether lower dose of intravenous acetaminophen still functions with comparable efficacy. It is important to address this question in that there might be a “ceiling effect” of acetaminophen, such that higher doses of acetaminophen do not provide greater benefit yet increase risk of hepatotoxicity (Hahn et al., 2003). In fact, the minimum useful single dose of the medication may be as low as 5 mg/kg (Hahn et al., 2003). Given the ceiling effect and the desire to minimize the risk of dose-related hepatotoxicity, the Chinese Center for Drug Evaluation now recommends limiting the daily dose of acetaminophen to 2 g for adults (Evaluation and Considerations regarding the dosage of acetaminophen, 2012). Therefore, we evaluated the efficacy and safety of intravenous acetaminophen administered in the first 24 h in patients who underwent orthopedic surgery at the dose of 500 mg, every 6 h. This regime reduced 24-h morphine consumption by 32.6% in the modified intention-to-treat analysis and 35.7% in the per-protocol analysis.

The efficacy of 2 g intravenous acetaminophen per day seems comparable to the efficacy of 4 g daily intravenous acetaminophen in decreasing the 24-h opioids consumption in orthopedic surgery with the range of decrease of 10–50.5% (5.76–19.1 mg) (Delbos and Boccard, 1995; Sinatra et al., 2005; Camu et al., 2017; Huang et al., 2018; Takeda et al., 2019). Takeda et al. (2019) demonstrated that intravenous acetaminophen in multimodal analgesia including femoral nerve block and intravenous fentanyl could reduce the dosage of opioids by 5.76 mg (10%) compared to the standard analgesic regimen. Camu et al. (2017) proved that intravenous acetaminophen could provide opioid-sparing efficacy of 9.95 mg (26.8%) compared to the placebo. In the study reported by Delbos and Boccard (1995), intravenous acetaminophen has demonstrated a morphine-sparing effect with 8.6 mg (20.0%) in postoperative orthopedic pain. More strikingly, Huang et al. reported that the overall opioid consumption in the intravenous acetaminophen group was noted to be 50.5% less than that observed in the placebo group in the multimodal analgesic regime including intra-articular joint injection and postoperative medication (Huang et al., 2018). However, the difference in our study did not reach the minimum clinically important difference (MCID) which was reported to be 10 mg for absolute reductions or 40% for relative reductions (Laigaard et al., 2021). The smaller dosage of intravenous acetaminophen, various sub-types of orthopedic surgeries, and differences in postoperative management perhaps contributed to the difference on the effect of intravenous acetaminophen in reducing opioids consumption in our study compared to other studies.

Moreover, we found that intravenous acetaminophen at a daily dose of 2 g did not significantly reduce pain intensity at rest or during movement. This finding is consistent with the conclusion (Guo et al., 2018) that perioperative intravenous acetaminophen can significantly reduce total opioid consumption but not average pain scores. One explanation may be that all patients have titrated PCA morphine until achieving comparable pain levels in the two groups. This would mean that the perioperative acetaminophen truly reduced the need for postoperative PCA morphine consumption in our study.

Three subjects in our acetaminophen group experienced severe postoperative adverse events: one suffered infection, another suffered scrotal hematoma and the third patient experienced reperfusion-induced injury. None of these serious adverse events was attributed to the use of acetaminophen. The reduced consumption volume of the opioids in the perioperative period would be expected to reduce the incidence of opioid-related adverse events. However, a significant difference was not observed in the current trial. 21.6% of patients in the acetaminophen group and 23.5% of patients in the placebo group reported nausea and vomiting (*p* = 0.70). Our result was also in accordance with a meta-analysis of studies analyzing the occurrence of postoperative nausea and vomiting after perioperative administration of intravenous acetaminophen in the population of knee or hip arthroplasty (Guo et al., 2018). Due to the different dosages among the studies, direct comparison could not be conducted between our results and the results of the studies from Belgium, France, Portugal, Spain, and Switzerland (29.6%) (Camu et al., 2017) and the US (38.7%) (Sinatra et al., 2005). However, our trial suggests that intravenous acetaminophen at a daily dose of 2 g does not significantly increase the risk of postoperative nausea and vomiting in patients undergoing orthopedic surgery. It remains to be investigated whether our regimen (intravenous acetaminophen 500 mg q6h) offers a better safety profile than regimens with higher doses and more frequent schedules.

Acetaminophen damages the liver in a dose-dependent manner, and it is the most common cause of acute liver injuries in the United States (Bower et al., 2007). In fact, such liver injuries can occur even at the therapeutic doses under certain conditions, such as the individual fasts or consumes alcohol (Whitcomb and Block, 1994). There was no obvious liver injure observed in our research. One patient in our trial experienced a transient increase in alanine aminotransferase and aspartate aminotransferase on the fourth day after surgery. It is noteworthy to point out that this patient also took cefoxitin, omeprazole and tramadol after the treatment period of acetaminophen, all of which may affect the result of liver function tests.

## 5 Strengths and limitations

Our study involved several large hospitals across China, and we strictly applied rigorous inclusion and exclusion criteria to minimize the influence of confounding factors. Since morphine was the only drug used for postoperative analgesia during the 24 h after the first administration of experimental medications, we were able to exclude interference from other analgesic agents on the consumption of 24-h opioids.

On the other hand, our trial has several limitations. First, the generalizability of our findings is jeopardized by the exclusive use of morphine for postoperative analgesia. In the clinic, a range of approaches are typically used, including other pain relievers and nerve blocks. Future work should explore the efficacy and safety of intravenous acetaminophen when combined with, for example, regional anesthesia or non-steroidal, anti-inflammatory drugs after orthopedic surgery. Second, due to the lack of treatment cost data in the trial, we were unable to analyze the cost-effectiveness of our acetaminophen regime. Third, several outcomes that are helpful to comprehensively evaluate the opioid-sparing effects of a postoperative medication were left untouched in our trial, such as sleep quality, occurrence of deep vein thrombosis, and decreased peak respiratory flow (Gewandter et al., 2021)., Fourth, the variety range of the patients enrolled in our trial was relatively narrow. We only conducted the study in adults who underwent orthopedic surgery under general anesthesia. Further studies enrolling other types of patients undergoing other surgeries should be conducted to further investigate the efficacy and the safety of acetaminophen as a postoperative opioid-sparing agent.

## 6 Conclusion

Administering intravenous acetaminophen at a dose of 500 mg every 6 h after orthopedic surgery resulted in a statistically significant, but not clinically meaningful decrease of 24-h morphine consumption in Chinese adults, without significantly increasing the risk of postoperative adverse effects.

## Data Availability

The original contributions presented in the study are included in the article/Supplementary Material, further inquiries can be directed to the corresponding authors.
