# Supplementary material for: Efficacy and safety of intravenous acetaminophen (2 g/day) for reducing opioid consumption in Chinese adults after elective orthopedic surgery: A multicenter randomized controlled trial
Source: Front Pharmacol. 2022 Jul 22;13:909572. doi: 10.3389/fphar.2022.909572 (PMC9355325; doi:10.3389/fphar.2022.909572)
Supplement: Supplementary file 1 [file DataSheet4.docx]

**Appendix IV**

**Pilot study**

In a phase II pilot trial, patients 18-70 years old who were scheduled for orthopedic, abdominal or gynecological surgery under general anesthesia were randomized 1:1:1 to receive 325 mg of intravenous acetaminophen (in 32.5 mL, starting 30 min prior to the end of surgery, then repeated every 6 h for 24 h), 500 mg of intravenous acetaminophen (in 50 mL), or placebo (normal saline) (36 patients per group). All patients used morphine for postoperative patient-controlled analgesia (concentration, 0.2 mg/ml; bolus, 1 mg; lock-out time, 5 min; background infusion, 1.25 ml/h). Per-protocol analysis across all types of surgery showed that 24-h morphine consumption did not differ significantly among the three groups (*P* = 0.22). Among patients undergoing orthopedic surgery, 24-h morphine consumption was lower in the 500 mg acetaminophen group than in the placebo control (11.6 ± 5.7 *vs.* 16.6 ± 11.2 mg; mean difference 5 mg). Safety outcomes did not differ significantly among the three groups.
