# Supplementary material for: Efficacy and safety of intravenous acetaminophen (2 g/day) for reducing opioid consumption in Chinese adults after elective orthopedic surgery: A multicenter randomized controlled trial
Source: Front Pharmacol. 2022 Jul 22;13:909572. doi: 10.3389/fphar.2022.909572 (PMC9355325; doi:10.3389/fphar.2022.909572)
Supplement: Supplementary file 2 [file DataSheet3.docx]

**Appendix III**

**Criteria for assessing whether adverse events are related to the study medication [1]**

*Definitely related:* the adverse event occurred at a reasonable time after medication and is consistent with known reactions to the study medication (i.e. improvement after drug withdrawal, repeat event after repeated administration).

*Probably related:* the adverse event occurred at a reasonable time after medication and is consistent with known reactions to the study medication. There is unlikely to be an alternative explanation, such as a concomitant medication or disease.

*Probably not related:* the adverse event did not occur at a reasonable time after medication and is inconsistent with known reactions to the study medication.

*Definitely not related:* the adverse event did not occur at a reasonable time after medication, and the reaction type conforms to the known non-experimental drug.

**References**

[1] Youyu Z. Guidelines for clinical research of New Chinese medicine: Chinese Medical And Technology Press, 2002. Available from: https://kdocs.cn/l/cerCyzLEQtR0
