# Supplementary material for: Efficacy and safety of intravenous acetaminophen (2 g/day) for reducing opioid consumption in Chinese adults after elective orthopedic surgery: A multicenter randomized controlled trial
Source: Front Pharmacol. 2022 Jul 22;13:909572. doi: 10.3389/fphar.2022.909572 (PMC9355325; doi:10.3389/fphar.2022.909572)
Supplement: Supplementary file 3 [file DataSheet2.docx]

**Appendix II**

**Study management**

Prior to the start of the clinical trial, the principal investigator at each study center provided standardized training to local researchers about the trial protocol and about principles of Good Clinical Practice. Researchers were instructed in standardized methods for recording data and assessing patients, and they were told to apply the trial protocol strictly. Clinical research associates regularly conducted on-site inspections of the study sites to ensure strict adherence to protocol and to check Case Report Forms against original data.
