# Supplementary material for: Efficacy and safety of intravenous acetaminophen (2 g/day) for reducing opioid consumption in Chinese adults after elective orthopedic surgery: A multicenter randomized controlled trial
Source: Front Pharmacol. 2022 Jul 22;13:909572. doi: 10.3389/fphar.2022.909572 (PMC9355325; doi:10.3389/fphar.2022.909572)
Supplement: Supplementary file 4 [file DataSheet1.docx]

**Appendix I**

**List of participating institutions**

1. Beijing Chao-Yang Hospital, Capital Medical University, Beijing, China
2. Chenzhou No. 1 People's Hospital, Chenzhou, China
3. First Affiliated Hospital, Xinjiang Medical University, Xinjiang, China
4. Liuzhou People's Hospital, Liuzhou, China
5. Ningbo No. 2 Hospital, Ningbo, China
6. Second Affiliated Hospital of Xi’an Jiaotong University, Xi’an, China
7. Shanghai Eastern Hospital, Shanghai, China
8. The First Hospital of Jilin University, Changchun, China
9. The Third Xiangya Hospital of Central South University, Changsha, China
10. Three Gorges Central Hospital, Chongqing, China
11. Tianjin First Central Hospital, Tianjin, China
12. Tianjin Medical University General Hospital, Tianjin, China
13. West China Hospital, Sichuan University, Chengdu, China
14. Wuxi No. 4 People's Hospital, Wuxi, China
15. Xuanwu Hospital, Capital Medical University, Beijing, China
16. Yuhuangding Hospital, Yantai, China
17. Zhongshan People's Hospital, Zhongshan, China

**The number of patients enrolled at each institution**

| Institution | Acetaminophen  (n=119) | Placebo  (n=121) | Total |  |
| --- | --- | --- | --- | --- |
| West China Hospital, Sichuan University | 13 | 13 | 26 | |
| Chenzhou No. 1 People's Hospital | 10 | 14 | 24 | |
| The First Hospital of Jilin University | 9 | 10 | 19 | |
| Liuzhou People's Hospital | 13 | 13 | 26 | |
| Ningbo No. 2 Hospital | 4 | 7 | 11 | |
| Beijing Chao-Yang Hospital, Capital Medical University | 6 | 4 | 10 | |
| Tianjin Medical University General Hospital | 6 | 6 | 12 | |
| Wuxi No. 4 People's Hospital | 11 | 6 | 17 | |
| The Third Xiangya Hospital of Central South University | 14 | 10 | 24 | |
| Tianjin First Central Hospital | 11 | 9 | 20 | |
| Yuhangding Hospital | 0 | 2 | 2 | |
| Zhongshan People's Hospital | 12 | 14 | 26 | |
| Three Gorges Central Hospital | 2 | 2 | 4 | |
| Shanghai Eastern Hospital | 4 | 4 | 8 | |
| Xuanwu Hospital, Capital Medical University | 2 | 3 | 5 | |
| Second Affiliated Hospital of Xi’an Jiaotong University | 1 | 2 | 3 | |
| First Affiliated Hospital, Xinjiang Medical University | 1 | 2 | 3 | |
